# Discovery of primary prostate cancer biomarkers using cross cancer learning

**DOI:** 10.1038/s41598-021-89789-x

**Published:** 2021-05-17

**Authors:** Kaiyue Zhou, Suzan Arslanturk, Douglas B. Craig, Elisabeth Heath, Sorin Draghici

**Affiliations:** 1 Department of Computer Science, Wayne State University, Detroit, 48201 USA; 2 Department of Oncology, Wayne State University, Detroit, 48201 USA; 3 Bioinformatics and Biostatistics Core, Barbara Ann Karmanos Cancer Institute, Detroit, 48201 USA; 4 Molecular Therapeutics Program, Barbara Ann Karmanos Cancer Institute, Detroit, 48201 USA; 5 Department of Obstetrics and Gynecology, Wayne State University, Detroit, 48201 USA

**Keywords:** Cancer genomics, Predictive markers

## Abstract

Prostate cancer (PCa), the second leading cause of cancer death in American men, is a relatively slow-growing malignancy with multiple early treatment options. Yet, a significant number of low-risk PCa patients are over-diagnosed and over-treated with significant and long-term quality of life effects. Further, there is ever increasing evidence of metastasis and higher mortality when hormone-sensitive or castration-resistant PCa tumors are treated indistinctively. Hence, the critical need is to discover clinically-relevant and actionable PCa biomarkers by better understanding the biology of PCa. In this paper, we have discovered novel biomarkers of PCa tumors through cross-cancer learning by leveraging the pathological and molecular similarities in the DNA repair pathways of ovarian, prostate, and breast cancer tumors. Cross-cancer disease learning enriches the study population and identifies genetic/phenotypic commonalities that are important across diseases with pathological and molecular similarities. Our results show that *ADIRF, SLC2A5, C3orf86, HSPA1B* are among the most significant PCa biomarkers, while *MTRNR2L1, EEPD1, TEPP* and *VN1R2* are jointly important biomarkers across prostate, breast and ovarian cancers. Our validation results have further shown that the discovered biomarkers can predict the disease state better than any randomly selected subset of differentially expressed prostate cancer genes.

## Introduction

Prostate cancer (PCa) is the second leading cause of cancer death in American men. PCa is generally a slow-growing malignancy with increased lead-time due to screening. Moreover, the efficacy of treatment options [e.g., surgery, radio-therapy, androgen deprivation therapy (ADT)] improved the median survival of patients and continue to evolve as treatment-related adverse effects are better defined^1^. However, some PCa tumors are aggressive (i.e. progressing from localized disease to metastasis) and are responsible for the majority of the prostate cancer associated mortalities. Hence, the identification of significant predictive biomarkers associated with primary and metastatic PCa would be critical in guiding the clinical decision-making. Furthermore, the cancer biomarkers can be used to measure the molecular pathway deregulations, which would justify the application of certain therapies, and customize treatment plans for individuals.

Past efforts in molecular biomarker discovery have been modestly successful and fell short in their ability to decisively contribute to PCa patient care mainly due to: (1) the lack of understanding of the pathobiology of cancer^2,3^; (2) underestimating the contribution of variants located in non-coding regions of genes^4^ and (3) lack of clinically relevant results due to issues in study design, assay platforms, and availability of specimens for biomarker development^5,6^. Besides, there is evidence that different cancers share similar genomic aberrations in the tumor cells which confirms the commonality in molecular mechanisms and biological functions. Hence, the discovery of significant predictive biomarkers among biologically similar cancers, regardless of the origins of tissue may shed light on some key alterations of carcinogenesis.

Recently, the US Food and Drug Administration (FDA) approved the first multi-cancer treatment (Keytruda), for patients whose cancers have a common specific biomarker. This is the first time that the FDA has approved a drug based on a common biomarker, instead of the organ the tumor has originated. Despite this, the majority of biomarker discovery studies consider each cancer disease in isolation from the rest, and attempt to characterize the phenotypes and discover influential biomarkers that are cancer-type specific. Hence, the critical need is to discover clinically relevant and actionable PCa biomarkers by better understanding the biology of PCa through the exploitation of cancers with similar molecular and genetic aberrations.

Oncologists have closely looked at ovarian and breast cancers and identified that the tumors arising from these cancers are typically hormone-dependent and have remarkable underlying pathological and molecular similarities to prostate cancer in their DNA repair pathway abnormalities^7^. Alterations in DNA repair genes are common in primary prostate cancer and metastatic, castration-resistant prostate cancer (mCRPC) through mutations or deletions in *BRCA2, BRCA1, CDK12, ATM, FANCD2*, or *RAD51C*^8–10^. Robinson et. al. discovered that 23% of mCRPC harbor DNA repair pathways aberrations^11^. In comparison, the prevalence of germline or somatic aberrations in genes involved in the DNA damage repair pathway is identified at 19%^12^. Similarly, mutations in *BRCA1, BRCA2, ATM, RAD51C* were found in patients with triple negative/basal-like and non-triple negative breast cancers^13^. Studies have further shown that the basal-like ovarian and breast cancer tumors had similar rates and spectrums of mutations in DNA repair genes^14–18^. These biological similarities have led to remarkably similar treatment options. For instance, combining the androgen deprivation therapy (ADT) with PARP inhibitors (i.e. drugs already used in breast cancer treatment) is shown to be an effective approach in reducing the progression and recurrence of prostate cancer^19^. Several single agent activity PARP inhibitors (PARPi) are recently approved for treating certain ovarian and breast cancers^19^.

In this paper, we have built a data-driven deep learning approach, referred to as cross-cancer learning for PCa biomarker discovery. Cross-cancer disease learning has great potential in terms of enriching the study population and identifying jointly important biomarkers and treatment options across biologically similar diseases. Traditional machine learning driven molecular data based biomarker discovery approaches fail to achieve satisfactory results when there is limited sample size. In addition, as in the case of advanced and lethal PCa, the class imbalance issues further inhibit the discovery of promising biomarkers. Several deep learning techniques, in contrast, extract knowledge from one or more similar tasks without restrictions on domains and distributions to enhance the learning process by enriching the study population and exploiting commonalities and differences across tasks. There have been numerous deep learning applications in different domains^20–24^, including bioinformatics^25,26^, and cancer imaging^27,28^. More recently, it has been successfully applied on cancer drug response^29,30^, unsupervised feature learning for cancer classification^31^ and semi-supervised learning for cancer classification^32^.

**Figure 1:**
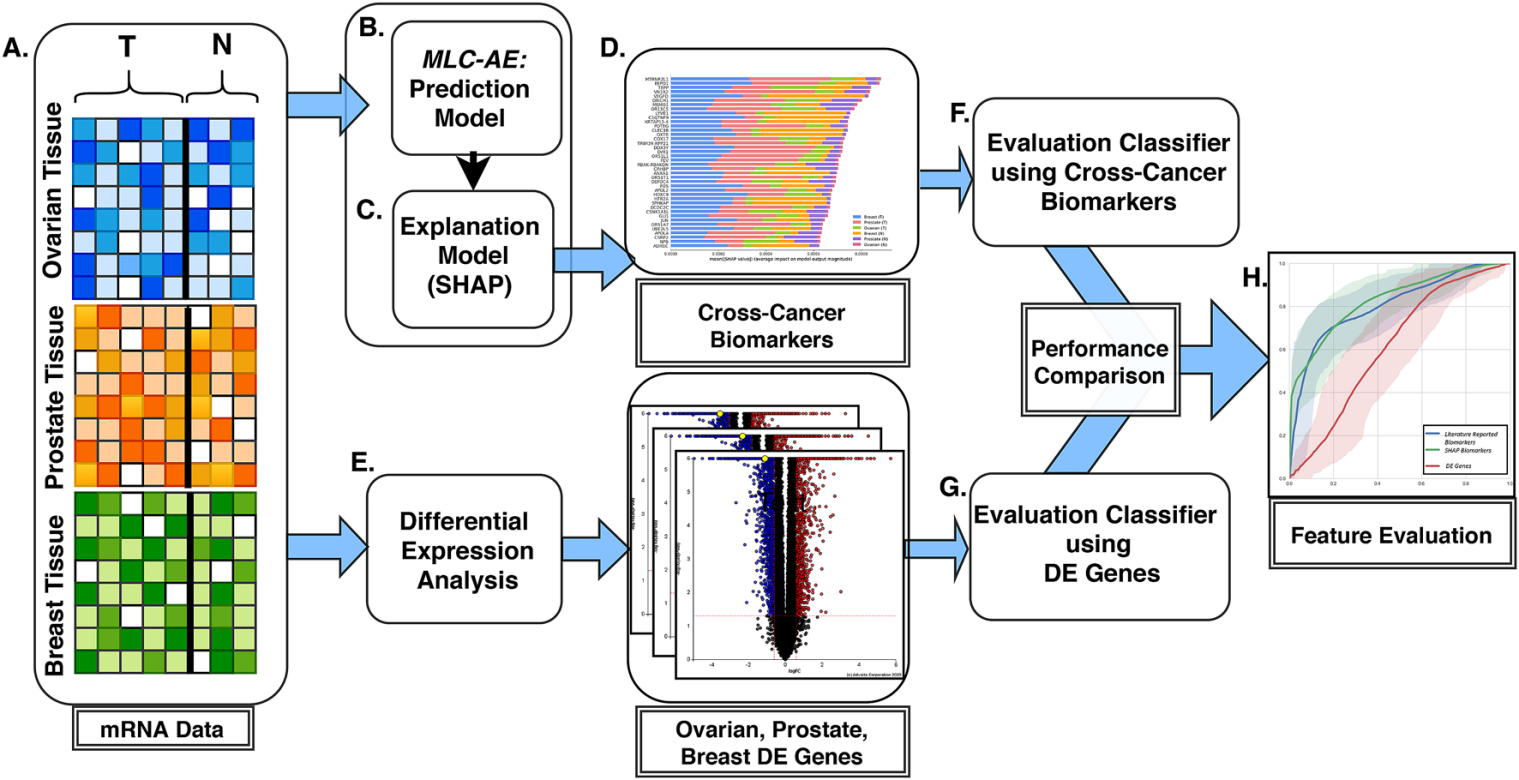
The proposed framework: (**A**) gene expression data from ovarian, prostate and breast tissues (*T*: tumor, *N*: normal). (**B**) A multi-label classification auto-encoder (MLC-AE) is built to predict the tissue type (ovary, prostate and breast) and disease state (tumor and normal) of a given gene expression profile. (**C**) An explanation model (SHAP) is used to identify the contribution of each input node (gene) towards prediction. (**D**) The SHAP values are used to rank the genes. (**E**) In parallel, a differential expression analysis is used to identify the DE genes associated with ovarian, prostate and breast cancers. An evaluation classifier is built using only cross-cancer biomarkers of prostate cancer (**F**), and using only a randomly selected subset of DE genes (**G**). Finally, the performances of the evaluation classifier using the cross-cancer biomarkers and random set of DE genes are compared (**H**).

## Method

Here, we have developed a cross-cancer learning approach to identify a clinically-relevant set of biomarkers associated with ovarian, prostate and breast cancers. Our framework for cross-cancer learning model development, biomarker discovery and evaluation is described in Fig. 1.

### Tissue type and disease state prediction

One limitation of molecular datasets in general is related to the limited number of samples and the high dimensional feature space, leading to the “curse of dimensionality”^33,34^. The issue of dimensionality is usually managed by feature selection or transformation techniques, leading inevitably to a loss of valuable predictive information. Extracting a meaningful low-dimensional latent representation from a molecular profile is the key to success in overcoming the problem of high-dimensional data with small sample size. For this purpose, our approach utilizes an autoencoder (AE) composed of a supervised deep learning architecture. The lower dimensional latent representation of the mRNA gene expression profiles is then used in a multi-label classification task (referred as MLC-AE) by exploiting commonalities and differences across tasks to differentiate the ovarian, prostate, breast tissues (the three tissues hereafter) and disease states (solid tumor vs. adjacent normal tissue).

**Figure 2:**
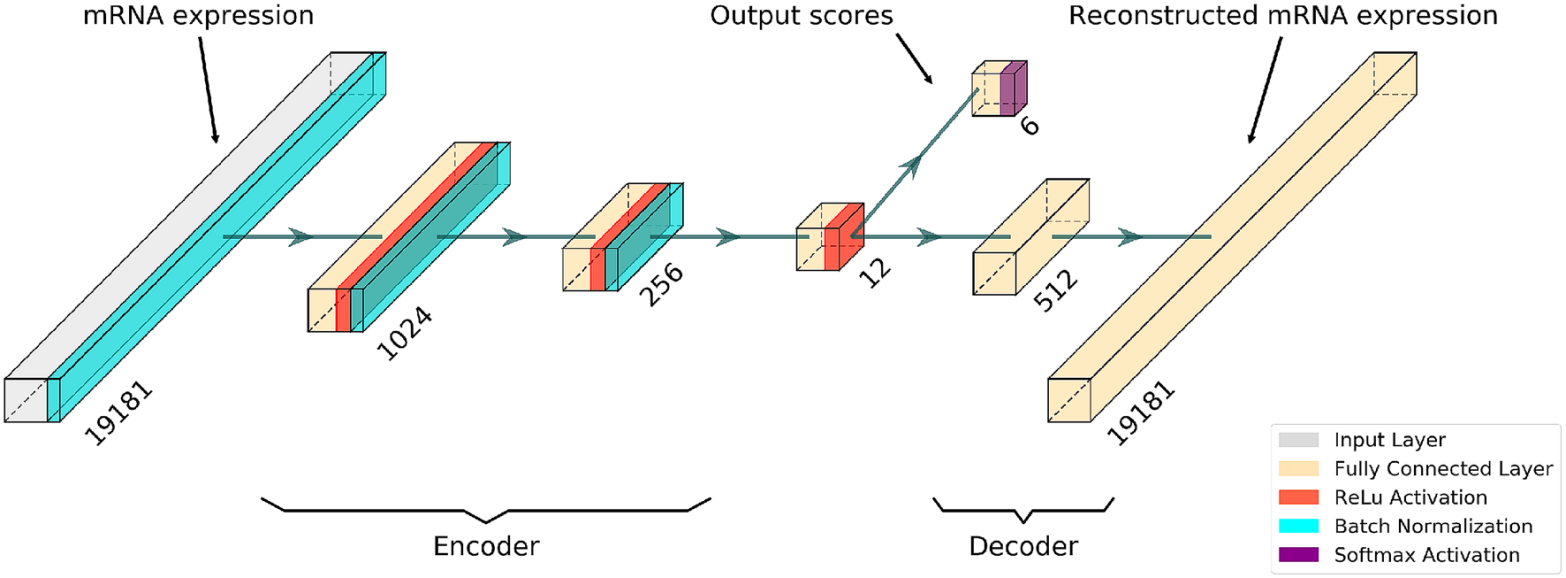
The network architecture: an autoencoder is utilized to extract a meaningful low-dimensional latent representation from a molecular profile. Meanwhile, the 12-dimensional latent representation has the ability to correctly classify the three tissues and disease states of a gene expression profile. Numbers aligning along each layer represent the number of nodes.

Azarkhalili et al.’s recent paper^35^ has inspired and given us the basis on which we have built our MLC-AE architecture as shown in Fig. 2. Note that our prediction model is different, as DeePathology^35^ uses two separate output layers for tumor and normal types. The encoder part of our model converts the mRNA expression profile of each sample to a lower dimensional latent space, and the decoder reconstructs an approximation of the same input with minimal loss. We utilized the cosine similarity as the loss for the classification tasks. The compressed latent representation is then able to represent all classes within our data.

### Biomarker discovery using explainable AI

While deep learning models reach impressive prediction accuracies, their nested non-linear structure makes them highly non-transparent, i.e., it is not clear what information from the input data makes them actually arrive at their decisions. For clinicians, these models appear as “black boxes” and hence hamper their confidence in using them for clinical decision making, mainly because they are unable to compare to and integrate their expert opinion with the predictions. Particularly important in this study is the ability to learn from the model and extract distilled biomarker information critical to PCa.

Several methods have been proposed in the literature to identify the importance of each feature. Kunpeng et al. introduced a reinforcement learning based approach by constructing a state vector using statistical analysis, autoencoders, or graph convolution networks^36^. However, obtaining the state vector requires to compute the correlation among features, which is unrealistic when the feature size is too large. DeepPINK proposed by Lu et al.^37^ requires to double the size of features for computing the original and knockoff features in the pairwise coupling layer to finally obtain the feature importance. However, this approach reduces the speed of computation and increases the architecture demands, potentially making the approach unfeasible for tens of thousand of variables. In this study, we have utilized the SHapley Additive exPlanations (SHAP)^38^, a game theoretic approach to explain and interpret the MLC-AE model. The framework proposed here uses SHAP values as a way to extract feature importance across three cancers. When a neural network model makes a certain prediction (e.g. predicting a sample to be a prostate tumor or normal tissue) based on a set of features (i.e. gene expressions), the SHAP method calculates the change in performance with and without the presence of each feature. Those features leading to a significant performance reduction with their absence will be assigned a higher contribution score. Given the high dimensional feature space ($\approx$ 19,000 genes for each sample), this procedure requires a substantial computational effort. To address this, we use the Gradient Explainer^39^ as a model-specific approximation of expected SHAP values.

After the prediction network is fully trained, the SHAP explanation model described above is utilized to assess the significance of each feature. Given a dataset with N features (i.e. genes), ${x_1, x_2, \ldots , x_N} \in X$, where $x_i\in {\mathbb {R}}^C$, our goal is to identify the most relevant features, such that $f(X) \approx f(X_k)$, where *f* is the prediction of model, and $X_k \subset X$ denotes the top k significant features.

We have assigned a contribution score to each feature using the following approach. Let *F* be the set of all features, and $S \subset F$ be a subset. The explanation model assigns an importance value $\phi _i$ to each feature by calculating the change in performance with and without the presence of each feature *i*. Thereafter a model $f_{S \cup \{i\}}$ with feature i being present and the model $f_S$ with that feature being absent are separately conducted, and the impact of feature *i* is calculated through the difference in the predictive output of the two models: $f_{S \cup \{i\}}(x_{S \cup \{i\}}) - f_S(x_S)$, where $x_S$ denotes the vector of feature values in set S. Let $S \subseteq F \backslash \{i\}$ denote the subset of features excluding feature *i*. The SHAP scores^40^ can then be calculated as follows:

$$\begin{aligned} \phi _i=\sum _{S \subseteq F \backslash \{i\}} \frac{|S|!(|F| - |S| - 1)!}{|F|!} [f_{S \cup \{i\}}(x_{S \cup \{i\}}) - f_S(x_S)], \end{aligned}$$

where $| \cdot |$ denotes the cardinality of a set.

In an effort to reduce the computational complexity, a model-specific approximation of the expected SHAP values is used. Let *g* be the explanation model, in which, the simplified input $x'$ is often used to represent the original input data: $x = h_x (x')$, such that $g(z') \approx f(h_x (z')) = f_x(z')$ whenever $z' \approx x'$. Hence the attribution of each feature can be explained by the following equation:

1 $$\begin{aligned} \phi _i (f, x) = \sum _{z' \subseteq x'} \frac{|z'|!(M - |z'| - 1)!}{|M|!} [f_x(z') - f_x(z' \backslash i)], \end{aligned}$$

where *M* denotes the number of simplified input features, $z' \backslash i$ denotes $z'_i = 0$. With the simplified input mapping, $h_x (z') = z_S$, where *S* is the non-zero set in $z'$ and $z_S$ has zero values for features not in *S*, the approximation can be made to speed up computation^38^:

2 $$\begin{aligned} f_x(z') = f(h_x (z')) \approx f([z_S, E[z_{\overline{S}}]]), \end{aligned}$$

where $\overline{S}$ is the set of feature not in *S*.

### Pathway analysis

In order to identify the significantly disrupted pathways from the discovered biomarkers, we have used the impact analysis^41,42^. The impact analysis considers not only the measured gene expression changes, but also the structure and dynamics of a signaling pathway. The perturbation accumulation can be calculated for each pathway as follows:

3 $$\begin{aligned} Acc(g_i )=PF(g_i )-\Delta E(g_i) \end{aligned}$$

where $\Delta E(g)$ represents the normalized measured expression change for all genes and PF(g) represents the perturbation factor for all genes on a given pathway $P_i$. The perturbation factor can be defined as follows:

4 $$\begin{aligned} PF(g) = \Delta E(g) + \sum _{u \in {US_g}} \beta _{ug} \frac{PF(u)}{N_{ds}(u)}. \end{aligned}$$

The second term takes the interactions between genes in a signaling pathway into account. The impact analysis calculates the sum of all perturbation factors of genes *u* directly upstream of the target gene *g*, normalized by the number of downstream genes $N_{ds}(u)$ and weighted by a factor $\beta _{ug}$ , that reflects the type of interaction: $\beta _{ug} = 1$ for activation, $\beta _{ug} = -1$ for repression. For a gene with no upstream genes, the perturbation factor, *PF*, will be the measured expression change $\Delta E(g)$. The impact factor of a pathway $P_i$ can then be calculated as follows:

5 $$\begin{aligned} IF(P_i) = log(\frac{1}{p_i}) + \frac{\sum _{g \in P_i} |PF(g)|}{|\overline{\Delta E}| \cdot N_{de}(P_i)}, \end{aligned}$$

where $p_i$ represents the probability of obtaining at least the observed number of differentially expressed (DE) genes, $N_{de}$, just by chance^41^.

## Experiments and results

### Datasets

Transcriptome profiles of 2180 samples with ovarian (OV), prostate (PRAD), and breast (BRCA) cancer tumors are obtained from the Genomic Data Commons (GDC) consortium, within The Cancer Genome Atlas (TCGA) database. The samples include solid tumors and adjacent normal tissue obtained through core needle biopsies. In order to enrich the sample size and population diversity, and alleviate the imbalance between labels, we have further integrated mRNA expression data from samples with prostate cancer tumors and adjacent normal tissue from data collected by Ren et. al.^43^ and Kannan et al.^44^. The data description is provided in Table 1.

**Table 1:** Data description and distribution of samples across training and testing.

Data source	Project	Primary site	Type	# of Genes	# of tumor samples	# of normal samples	# of samples	
							Training set	Testing set
GDC	TCGA-OV	Ovary	Transcriptome profiling	19212	371	0	327	44
	TCGA-PRAD	Prostate gland	Transcriptome profiling	19212	495	52	465	82
	TCGA-BRCA	Breast	Transcriptome profiling	19212	1091	113	1043	161
BioProject	Ren et al.	Prostate gland	Transcriptome profiling	19252	14	14	22	6
BioProject	Kannan et al.	Prostate gland	Transcriptome profiling	19252	20	10	23	7
				Total	1991	189	1880	300

The data from all three sources were TPM normalized. After considering the available gene IDs common across all data sources, 19,181 common genes were identified for further analysis.

### Prediction performance of MLC-AE

We used the approach described above to identify a set of clinically relevant biomarkers associated with primary prostate cancer using cross-cancer learning. The set of samples within each project is split into training and test sets (Table 1) and the MLC-AE model is built using only the samples in the training set. The fully trained model is then applied on the samples in the testing set for validation. Our model differentiated the tissue type and disease states with a 94% balanced accuracy on the validation set using cross-cancer learning compared to the 54% balanced accuracy using only the PCa samples. Balanced accuracy is calculated by normalizing the number of correctly predicted samples of each class by the class size.

**Figure 3:**
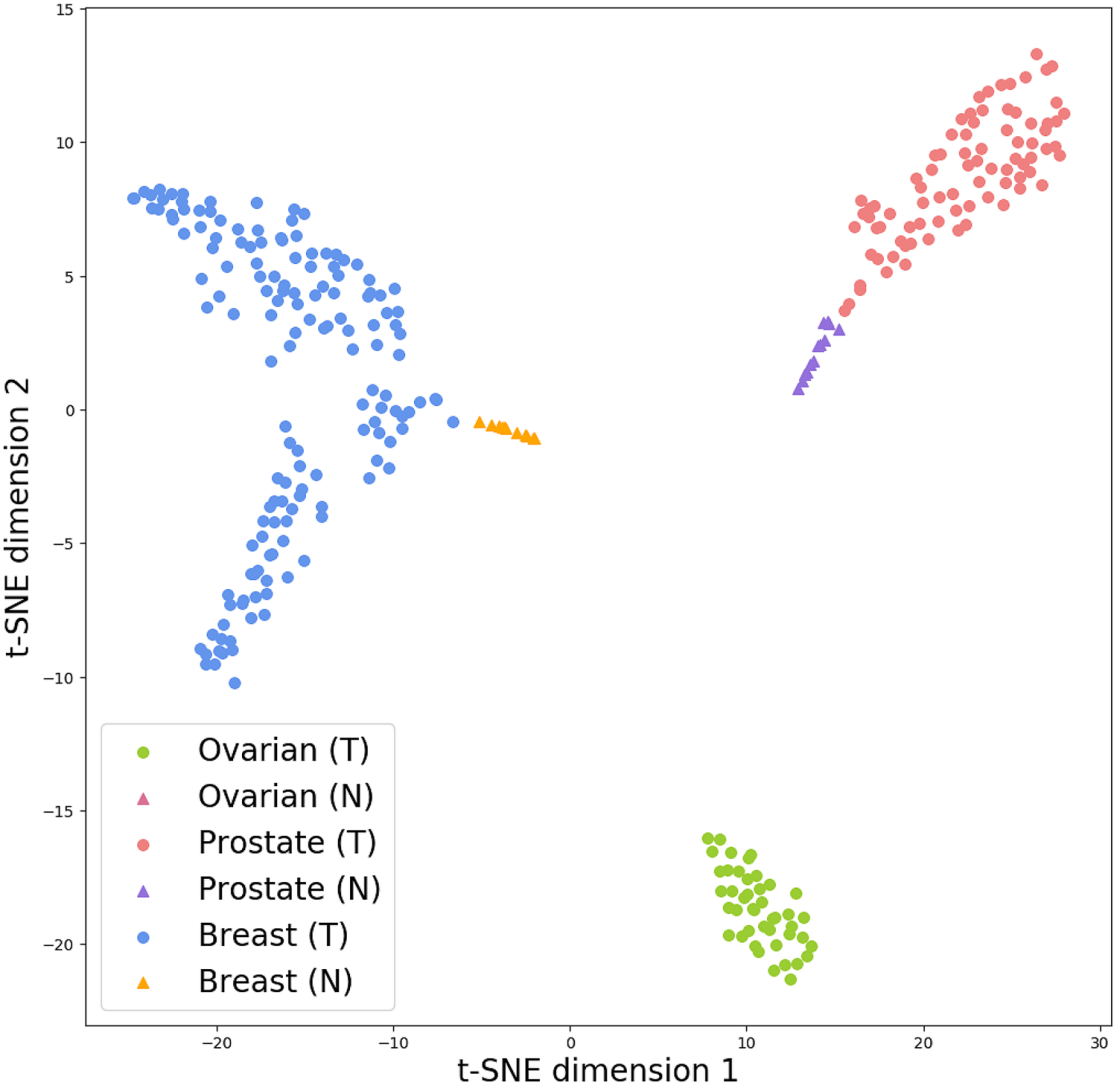
The T-SNE analysis using the 12 dimensional latent representation from our network can generally separate the classes into distinguishable clusters.

We further show that the 12-dimensional latent space encoded through the autoencoder has a discriminative dimension reduction as each tissue type and disease state is well-separated when plotted on the two dimensional space through the top principal components using the t-SNE plot as shown in Fig. 3.

### Biomarker discovery

**Figure 4:**
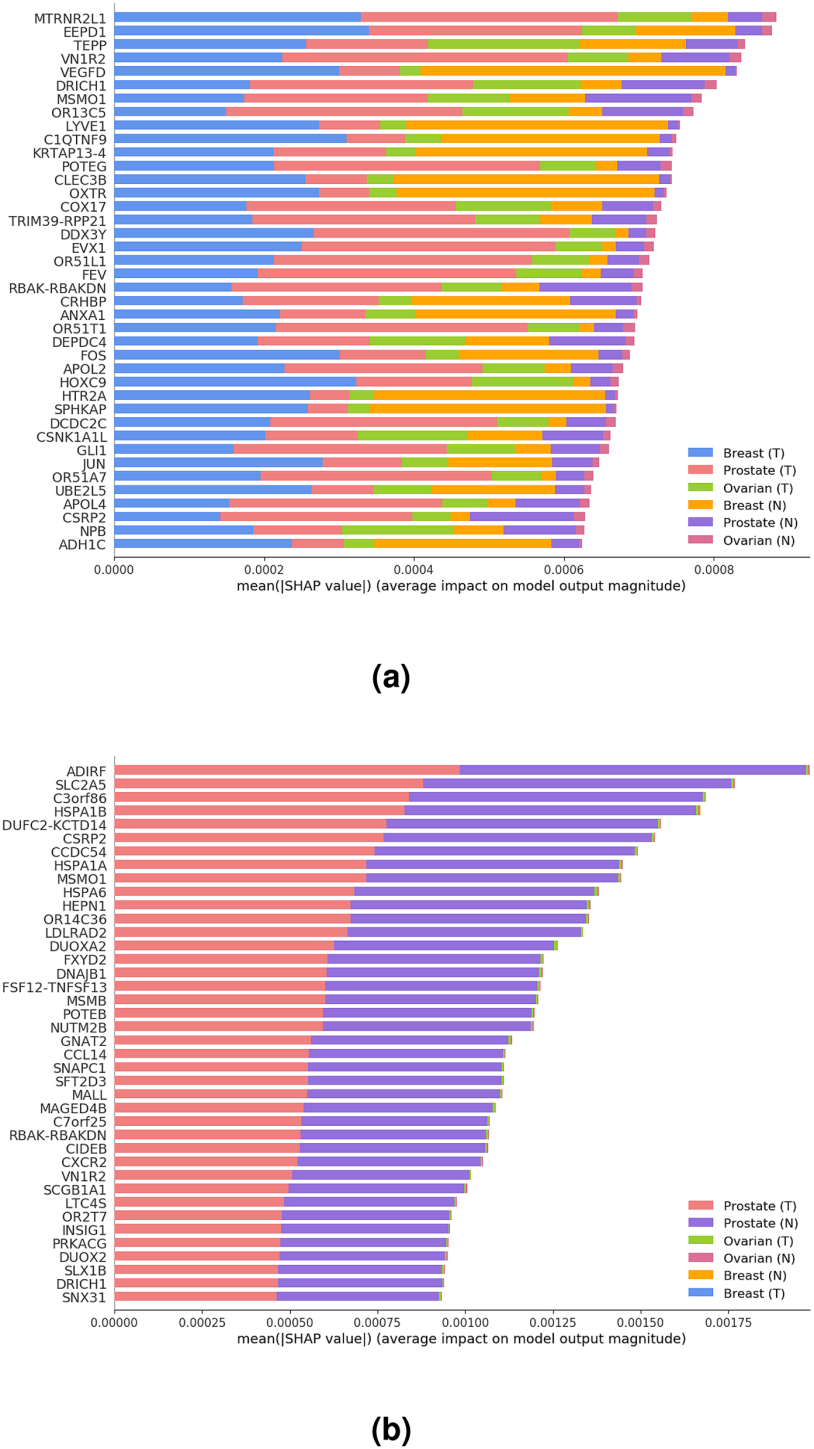
The most relevant genes identified by SHAP ordered based on (**a**) the total contribution scores of the three tissues and disease states (Top CC ALL), where (T) denotes tumor and (N) denotes normal, and (**b**) contribution scores identified solely based on the prostate tissue and its disease state (Top CC PR). Note that the gene-specific contribution scores (x-axis) are not directly comparable across (**a**) and (**b**), as each gene’s score is based on a normalized calculation where the total contribution score across all genes ($\approx$ 19 K) sums up to 1.

Here, we have used the SHAP method combined with the gradient explainer described above to identify the cross-cancer biomarkers with the highest contribution scores towards prediction. Figure 4a shows the most significant genes ranked based on their total contribution scores obtained from the three tissues combined (Top CC ALL) using the explanation model, and Fig. 4b shows the ranking based on the contribution scores obtained solely from prostate tissue (Top CC PR) using the same explanation model.

### Validation of discovered biomarkers

**Figure 5:**
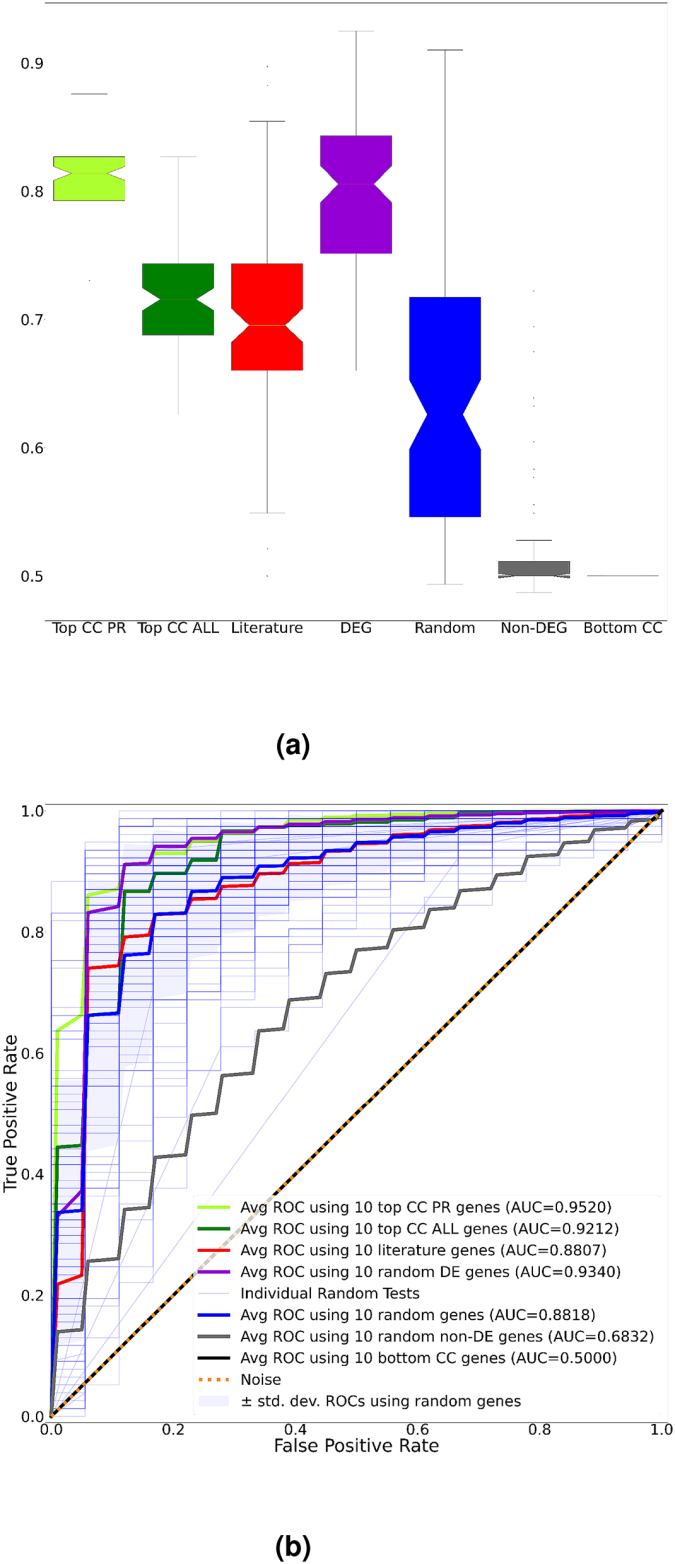
(**a**) From left to right, balanced accuracies of the evaluation classifier using a randomly selected set of the most relevant 10 Top CC PR, 10 Top CC ALL Biomarkers (out of top 40), randomly selected 10 PCa biomarkers reported in the literature (literature), randomly selected 10 DE genes (DEG), any randomly selected 10 genes (random), randomly selected 10 non-DE genes (non-DEG) and the least relevant 10 cross-cancer biomarkers (bottom CC). Reported results are across 100 independent runs of respective gene subset selections. The most relevant cross-cancer biomarkers (Top CC PR) are the most reproducible as seen from the narrow interquartile ranges. The lower performance reported using biomarkers identified from the literature are mainly due to several of them being non-DE in the datasets used. (**b**) The ROC curves of different gene subset selections. Note that, the Top CC PR and Top CC ALL show similar predictive performances. Meanwhile, the DE genes perform slightly lower than CC biomarkers. As expected, the genes with SHAP scores = 0 have no impact on prediction.

In order to assess the significance of the biomarkers identified, we constructed an evaluation classifier (i.e. a separate artificial neural network) by feeding only the significant biomarkers identified in the previous step as input to observe their predictive capability in differentiating samples with prostate tumor from adjacent normal tissue. Results of the analysis are summarized in Fig. 5a,b, which shows a comparison of the predictive performances using the discovered cross-cancer biomarkers, randomly selected subsets of DE genes and non-DE genes and additional biomarkers identified by consulted subject-matter experts and literature reviews (namely *HNF1B, KLK2, MYC, NFE2L2, POU5F1B, PTEN, RNASEL, SLC45A3, SOX2, SRD5A2, BRCA1, BRCA2, HOXB13, TP53, RAD51D, PALB2, NCOA3, MSR1, MSH2, MLH1, AIG1, ATM, BRAF, CDK12, CDKN1B, CHEK2, ELAC2, HIF1A*).The classifier was trained using only the training data and its performance was assessed only on the testing data. Results clearly show that the Top CC PR biomarkers are both superior in terms of their evaluation performance to any subset of DE genes as well as to the set of genes reported in the literature. Note that the average prediction performance obtained by randomly selected subsets of PCa DE genes have outperformed the Top CC ALL biomarkers. This is mainly due to the fact that biomarkers are validated based on their ability to distinguish prostate tumors from normal tissue. Given that the Top CC ALL biomarkers are those genes responsible for breast, prostate and ovarian cancers combined, their ability to solely predict the disease state of PCa patients is lower than top PCa genes.

**Figure 6:**
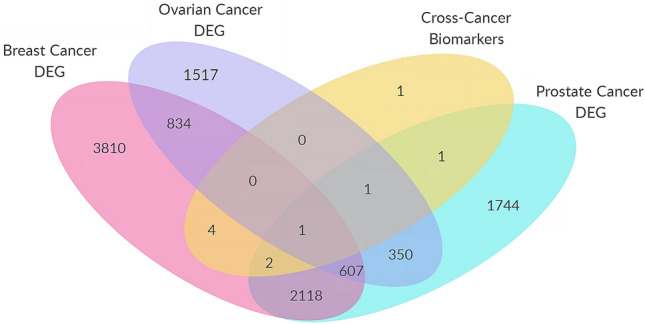
Venn diagram showing the relationship of the Top-10 cross-cancer biomarkers (Top CC ALL) and DE genes from the three cancers. All but one of the Top-10 cross-cancer (Top CC ALL) biomarkers are found to be differentially expressed.

Figure 6 shows a Venn diagram illustrating the relationship of the 10 cross-cancer biomarkers and DE genes from the three cancers identified through linear models analysis (limma). Threshold parameters used for the analysis are an absolute fold change greater than 0.6 and false discovery rate (FDR) adjusted p value less than 0.05. Note that, the only non-DE cross-cancer biomarker is reported to have a fold-change of 0.5 and a p value < 0.05. Results show that the cross-cancer learning is able to identify some non-DE PCa genes with predictive capability and several other biomarkers (4 out of top 10) are reported to be DE for breast cancer. This suggests that cross-cancer learning can also overcome the limitations of preset thresholds utilized in DE gene detection through its threshold-free nature. Results have further shown that top CC PR biomarkers (AUC = 0.95) appears to predict the disease-state better than any randomly selected DE PCa genes (AUC = 0.93) as seen in Fig. 5a. This suggests that cross-cancer approach has the potential to prioritize DE genes based on their phenotype associations rather than other correlations (e.g., such as the complex immune response to cancer). Further, the cross-cancer learning’s ability to prioritize DE genes can alleviate challenges in the investigation of personalized treatment options and drug repositioning.

To further assess the statistical significance of the cross-cancer biomarkers, we repeatedly selected a random set of genes, trained and tested our predictive model based on the selected genes and calculated the AUC distribution based on 400 runs. We computed the p value as the percentage of the random AUCs higher than the observed, and have shown a statistically significant (< 0.00001) improvement using the discovered top CC PR biomarkers.

To increase the diversity of our data and alleviate the imbalance between tumor and normal samples, we add two small data sources^43,44^ into our experiments. This causes heterogeneity as all data sources might have different distributions. For this reason, we conducted a new and independent experiment using the Affymetrix oligonucleotide arrays of 128 samples (63 normal prostate tissue adjacent to tumor, 65 primary prostate tumor) from GEO GDS2545 dataset. This dataset contains expression values of only 9,467 genes in which 17 out of our top 40 CC genes (in Fig. 4b) were present (6 out of our top 10). As such, we trained an independent classifier with 108 samples and test on the rest. This classifier uses the 17 top CC genes and 17 from random selection (for 100 times), respectively. Although the platforms used to measure the expression levels of genes are different (RNA-Seq vs. Microarray Gene Expression), our reported biomarkers are still able to identify the disease state significantly better (AUC = 0.84) than any randomly selected set of genes (AUC = 0.62) on this previously unseen dataset.

In the following section, the novel biomarkers discovered through cross-cancer learning are further investigated to measure associated pathway deregulations.

### Identification of significantly impacted pathways

Due to inherent bias present in individual studies, independent studies of the same disease often yield completely different lists of differential expressed genes, making interpretation extremely difficult^45^. Because of this, an important capability is related to the analysis of molecular mechanisms and signaling pathways associated with the cross-cancer biomarkers. As signals propagate through a given pathway, the specific subset of biomarkers may change continuously, on various time scales. However, the impacted pathways may remain the same.

**Figure 7:**
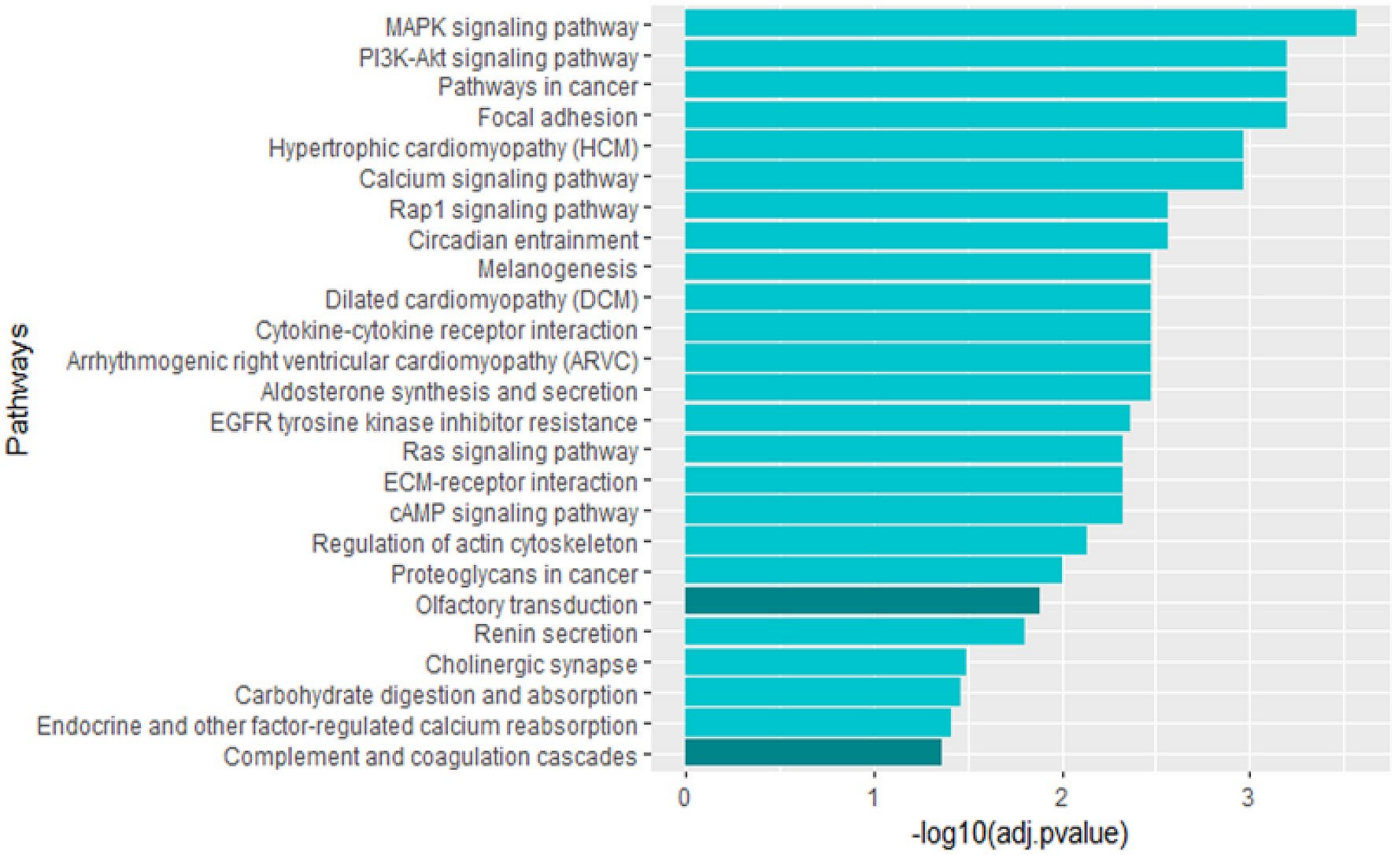
Molecular pathway deregulations measured using differentially expressed genes with highest contribution scores. Highlighted pathways are identified as significant using the cross-cancer genes, and not significant if one considers only DE genes.

In order to identify the significantly disrupted pathways in a given phenotype, we have used the impact analysis. Impact analysis considers not only the measured gene expression changes, but also the structure and dynamics of a signaling pathway. The fold-changes of the most relevant 1000 cross-cancer biomarkers that are differentially expressed are used to calculate the pathway deregulations. The significantly impacted pathways identified through impact analysis are shown in Fig. 7. The p values (x-axis) represent a combination of enrichment and perturbation p values corrected with FDR.

The highlighted pathways, (i.e. olfactory transduction and complement and coagulation cascades) are not identified as significant if analysis are conducted using only PCa DE genes. Hence, the identification of cross-cancer biomarkers also led to the discovery of several novel pathway deregulations common across the three cancers. These results are supported by the associations reported in the literature between those pathways and several cancers. In particular, significant associations between olfactory receptors (OR) transcript abundance and several cancers including large invasive breast carcinoma, and prostate cancer are reported^46–49^. The complement system, on the other hand, is considered as a component of immunity against invading pathogens and an imbalanced complement activation have been demonstrated in many types of tumors^50^. Several studies have reported the complement system’s role in tumour immunity and its therapeutic potential for ovarian cancer immunotherapy^51,52^.

**Figure 8:**
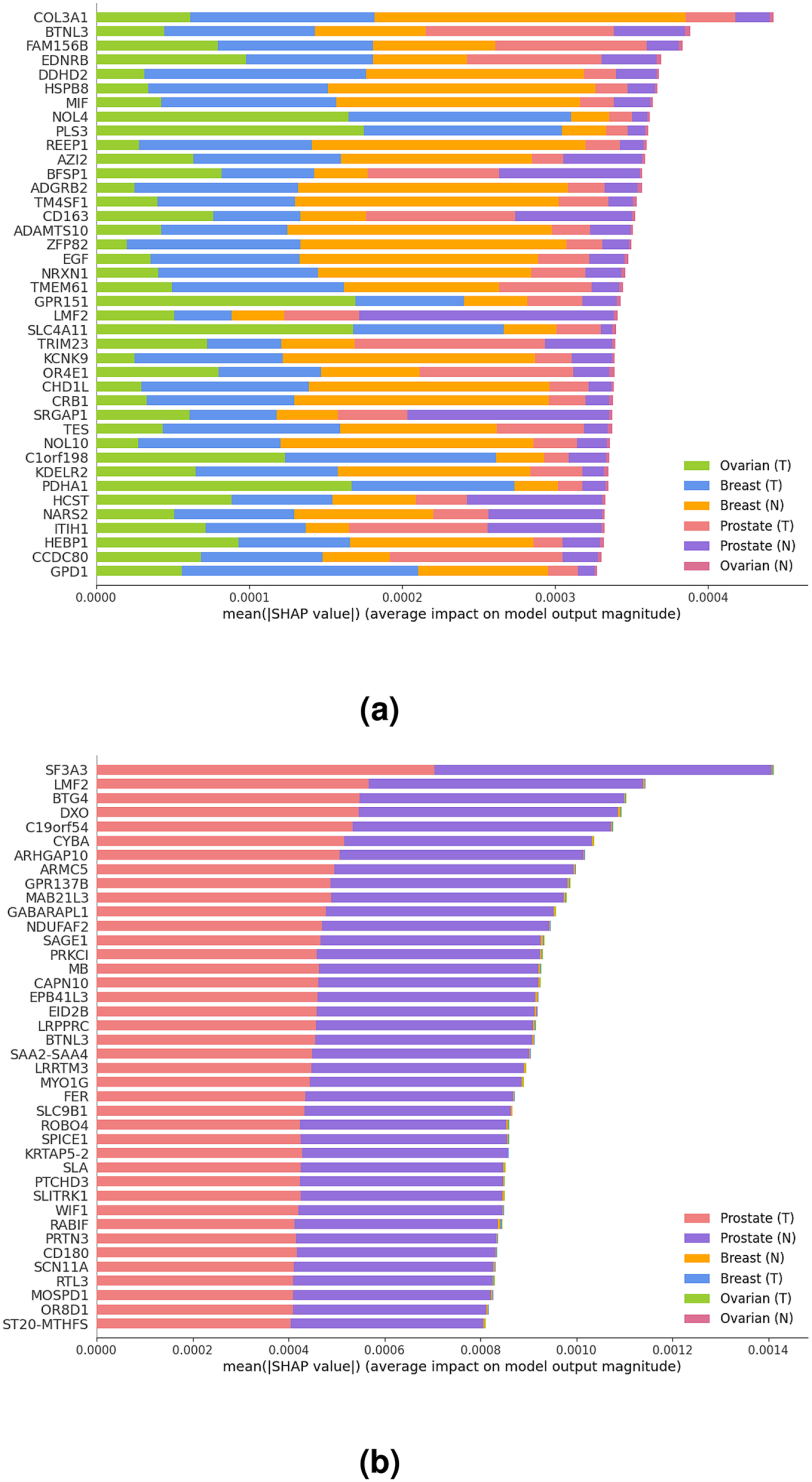
The most relevant genes for patients with advanced PCa identified by SHAP ordered based on (**a**) the total contribution scores of the three tissues and disease states (top CC ALL), and (**b**) contribution scores identified solely based on the prostate tissue and its disease state (top CC PR).

**Figure 9:**
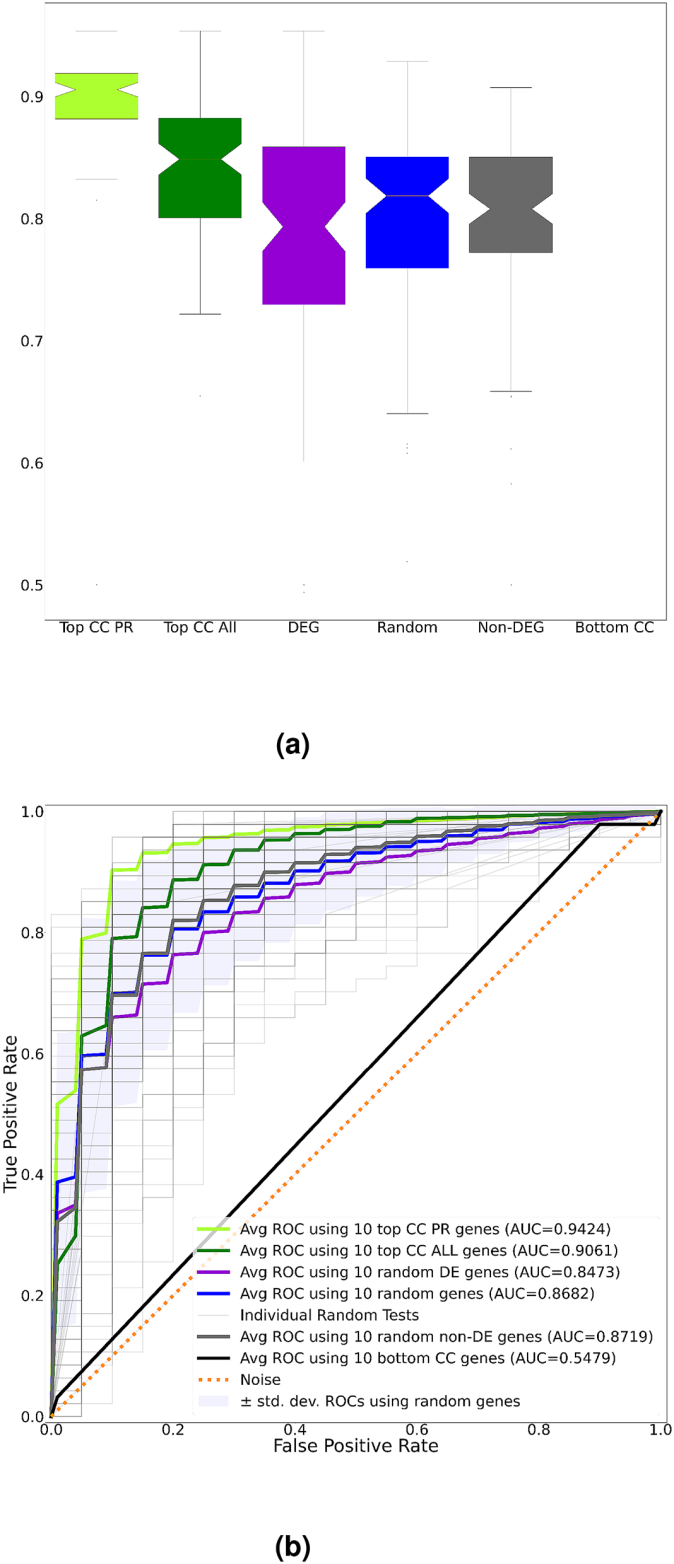
(**a**) From left to right, balanced accuracies of the evaluation classifier using a randomly selected set of the most relevant 10 Top CC PR, 10 Top CC ALL Biomarkers (out of top 40), randomly selected 10 DE genes (DEG), any randomly selected 10 genes (random), randomly selected 10 non-DE genes (non-DEG) and the least relevant 10 cross-cancer biomarkers (bottom CC). Reported results are across 100 independent runs of respective gene subset selections. Note that, CC biomarkers still outperform other gene subset selections. (**b**) The ROC Curves of different gene subset selections.

### Biomarker discovery of advanced prostate cancer

In this section, we refined the analyses discussed above, to discover biomarkers associated with more advanced forms of prostate cancer using mRNA gene expression levels. Here, an advanced case is defined as (1) a new tumor event through local recurrence, or distant metastasis, (2) biochemical evidence of disease (elevated PSA levels) after complete remission or response or (3) death due to cancer, all within 5 years of initial diagnosis.

Using TCGA data, we have extracted 640 advanced cancer samples from all three tissues combined, 139 of which have PCa. The PCa samples are then splitted into 72 (40 tumor and 32 normal) and 67 (47 tumor and 20 normal) samples for training and validation, respectively. The evaluation results have demonstrated a 89.15% balanced accuracy in differentiating advanced PCa samples from the adjacent normal tissues. Details of the cross-cancer biomarkers associated with advanced PCa and evaluation performances are shown in Figs. 8 and 9, respectively. Note that, the results for random subsets of genes (random) and non-DE genes (non-DEG) are possibly over-estimating their actual performance mainly due to: (1) the lack of reproducibility on differential expression analysis on limited sample sizes leading to a potentially misleading set of non-DE genes, and/or (2) limitations on model convergence due to insufficient samples and high dimensionality (small n, large p).

## Conclusion

In this paper, we proposed a data-driven deep learning approach, referred to as cross-cancer (CC) learning for PCa biomarker discovery. We have shown that the set of cross-cancer biomarkers have the ability to better distinguish the tumor from normal tissue than any other subset of genes using samples with primary prostate cancer. We have subsequently performed a biomarker-driven pathway analysis to better understand novel molecular mechanisms and pathway deregulations associated with biologically similar cancers.

One limitation and potential future work is to address the heterogeneity within the same disease by investigating the similarities and differences across ovarian, prostate, and breast cancer subtypes. For instance, the hormonally driven breast cancer subtypes Luminal A ($ER+/PR+$) and Luminal B ($ER+/PR-$) are known to have remarkable biological similarities with PCa. Similarly, several genomic features (BRCA1 inactivation, RB1 loss and cyclin E1 amplification, high expression of AKT3, MYC amplification and high expression; and a high frequency of TP53 mutations) were found to be similar between Basal-like breast cancer and high-grade serous ovarian cancer^53^. Given that, the molecular and histological subtypes of diseases should be investigated to better understand jointly important biomarkers across biologically similar diseases.

A set of clinically-relevant and reproducable biomarkers jointly important across different types of cancers have the potential to be utilized in the discovery of novel pharmaceutical cross-cancer treatments that target patients who respond poorly to organ-specific treatments. Future work involves developing a biomarker-driven analysis technique, using the cross-cancer biomarkers, that is able to support PCa drug-repurposing capabilities. This will help identifying and prioritizing several FDA-approved drugs, drugs under trial, or other chemicals that have a therapeutic effect by impacting the same pathway(s) in an antagonistic manner.

## Data Availability

The results published here are in whole or part based upon data generated by The Cancer Genome Atlas managed by the NCI and NHGRI. Information about TCGA can be found at http://cancergenome.nih.gov. The analysis source codes are available at https://github.com/ky-zhou/CCL-Discovery.
